# Sequential conversion from line defects to atomic clusters in monolayer WS_2_

**DOI:** 10.1186/s42649-020-00047-2

**Published:** 2020-11-30

**Authors:** Gyeong Hee Ryu, Ren-Jie Chan

**Affiliations:** 1 School of Materials Science and Engineering, Gyeongsang National University, Jinju, 52828 Republic of Korea; 2 Department of Materials, University of Oxford, 16 Parks Road, Oxford, OX1 3PH UK

**Keywords:** Cluster, Line defect, Hole, ADF-STEM, WS_2_

## Abstract

Transition metal dichalcogenides (TMD), which is composed of a transition metal atom and chalcogen ion atoms, usually form vacancies based on the knock-on threshold of each atom. In particular, when electron beam is irradiated on a monolayer TMD such as MoS_2_ and WS_2_, S vacancies are formed preferentially, and they are aligned linearly to constitute line defects. And then, a hole is formed at the point where the successively formed line defects collide, and metal clusters are also formed at the edge of the hole. This study reports a process in which the line defects formed in a monolayer WS_2_ sheet expends into holes. Here, the process in which the W cluster, which always occurs at the edge of the formed hole, goes through a uniform intermediate phase is explained based on the line defects and the formation behavior of the hole. Further investigation confirms the atomic structure of the intermediate phase using annular dark field scanning transition electron microscopy (ADF-STEM) and image simulation.

## Introduction

A formation of structural defects in two dimensional (2D) materials affects their intrinsic properties (Topsakal et al. 2008; Faccio and Mombrú 2012; Han et al. 2015). Various types of defects have been studied, like zero dimensional (0D) defects (Komsa et al. 2002; Azizi et al. 2014) and one dimensional (1D) defects (Lahiri et al. 2010; Botello-Mendez et al. 2011; Liu et al. 2012; van der Zande et al. 2013; Zhou et al. 2013; Enyashin et al. 2013; Lin et al. 2015; Barja et al. 2016). Defect theories of monolayer TMDs such as MoS_2_ and WS_2_ (Radisavljevic et al. 2011; Nourbakhsh et al. 2016; Liu et al. 2016) sheet with semiconducting properties for electronic and optoelectronic devices have been established, which explains that S atoms are easier to eject than W and Mo atoms. S loss leads to increasing vacancies and line defects that changes electrical properties, and as the width and length of the line defects increase, the transition from semiconductor to metallic properties (Ryu et al. 2016; Wang et al. 2016).

Generally, the large number of vacancies formed in a material are energetically disadvantageous, so they do not exist individually and tend to agglomerate together (Smallman and Bishop 1999). In two dimensional (2D) materials composed of two elements, holes with different aspects such as shape and edge termination are formed depending on knock-on thresholds of the materials (Ryu et al. 2016; Ryu et al. 2015; Park et al. 2015; Girit et al. 2009; Bieri et al. 2009; Farimani et al. 2014; Kotakoski et al. 2010). For edges of holes, they are similar to the hole surfaces in bulk materials, and linear defects including dislocations, grain boundary, and line defects are limited to 2D. In a case of monolayer hexagonal boron nitride, boron vacancies have been observed to diffuse and merge into extended triangular holes to reduce the surface energy of a larger hole. (Ryu et al. 2015; Alem et al. 2009; Alem et al. 2011). For a monolayer MoS_2_, the increasing density of S vacancies develops into long line defects and extended holes with MoS nanowires (Liu et al. 2013; Sang et al. 2018) and Mo clusters (Ryu et al. 2016).

Previous works in TMDs have demonstrated that chalcogen atom vacancies are easily formed and transition metal atoms tend to aggregate at edges of extended holes (Chen et al. 2018; Komsa et al. 2013; Le et al. 2014). However, it is relatively difficult to form line defects than WS_2_ due to the high energy barrier of S vacancy migration in MoS_2_. Here, we report details on the conversion process from line defects into W clusters through a crystalline intermediate phase in a monolayer WS_2_ sheet. ADF-STEM images are used to study atomic dynamics and are acquired in a clean area of WS_2_ to observe the atomic dynamics of the overall formation process.

## Results and discussion

**Fig. 1:**
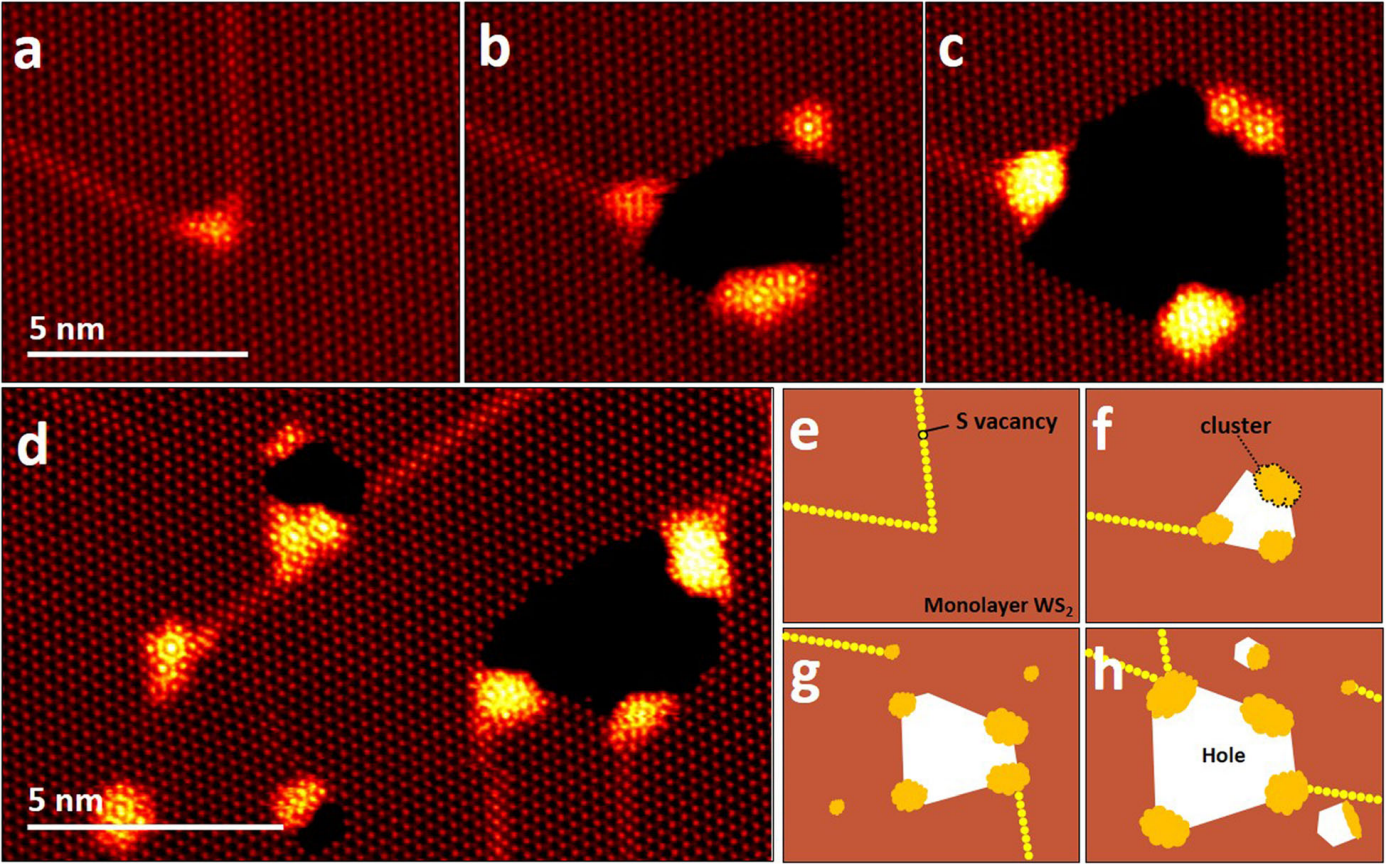
**a**–**c** ADF-STEM images showing the formation of atomic clusters at edges of a hole in the monolayer WS_2_. **d** ADF-STEM image showing extended holes with line defects and clusters. **e**–**h** Simple successive schematics showing a whole process for the formation of clusters and holes from line defects in the monolayer WS_2_ sheet

If an electron beam is irradiated onto a synthesized monolayer WS_2_ sheet using an acceleration voltage of 80 kV, long line defects are generally formed, and holes are formed at the point where the line defects collide (Fig. 1a and b). The knocking-off rate of S atoms is getting high as the hole is extended (Fig. 1c). This is because the S vacancies are linearly aligned to form the line defects and rapidly migrate to the exteneded hole. In addition, the line defects are often atomically uniform with a periodic lattice structure and they are absorbed into the hole leaving small clusters at edges of the holes (Fig. 1d). ADF-STEM images are used to investigate the exact atomic dynamics of the formation mechanism on the clusters. As shown in Fig. 1e-f, accumulated S vacancies are directly related to the formation of line defects, and when the line defects collide, topological holes are formed, leaving clusters at the edge of the holes.

**Fig. 2:**
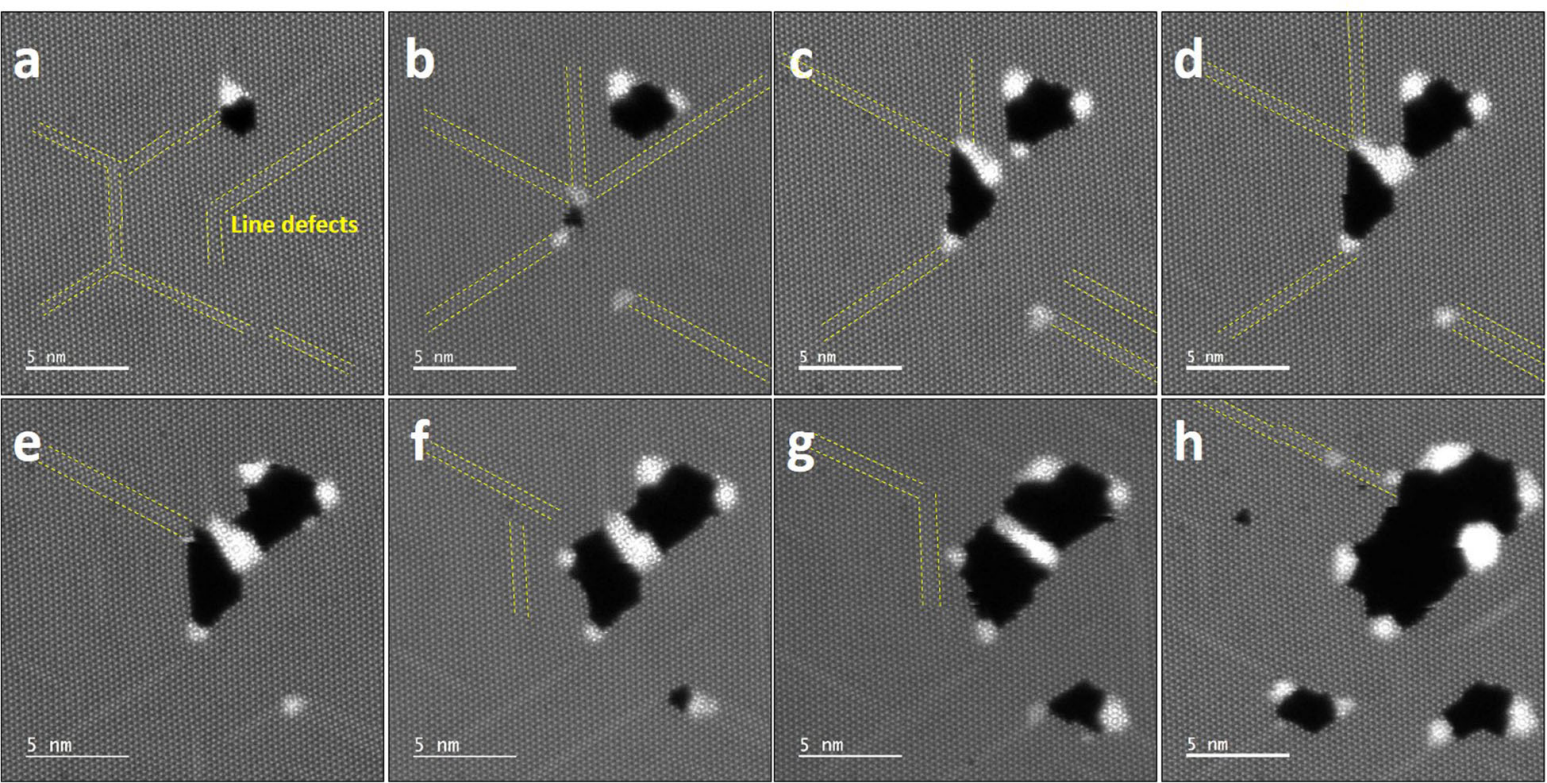
Successive ADF-STEM images showing the formation of clusters initiated from line defects in the monolayer WS_2_. Yellow dashed lines indicate long line defects

When a prolonged electron beam irradiates on the WS_2_ sheet, holes are formed in the WS_2_ due to the high concentration of S vacancies. The initiation point for the hole formation usually occurs at the intersection of long line defects and W clusters are formed at the edge of the hole or at the ends of the line defects. Figure 2 shows successive images of colliding line defects (Fig. 2a and b), and then a hole begins to open and extend (Fig. 2c−h). In Fig. 2h, the large hole absorbs the long line defects on the left, and the remaining line defects relate to the newly formed line defects. Once the hole is opened (Fig. 2c), the W cluster is adjusted, binding to the edge of the hole, and the hole also rapidly extends. Newly formed line defects near the existing holes are also absorbed by the hole when connecting and the hole is extended. The WS_2_ sheet surrounding the holes is still basically a pristine lattice because S vacancies quickly diffuse into the holes. W clusters are attached to the edges of the holes with bright contrast.

**Fig. 3:**
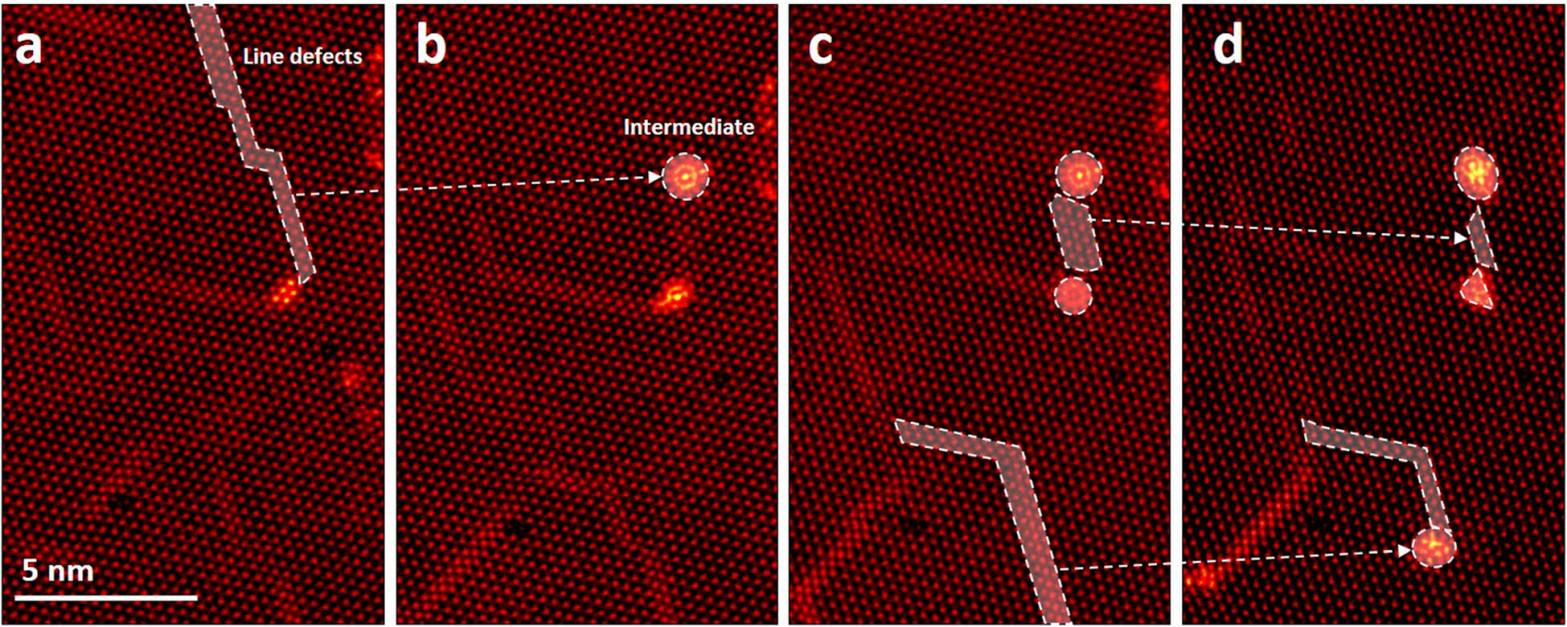
Formation of the intermediate phase of the atomic cluster inside the monolayer WS_2_ sheet. **a** ADF-STEM image showing line defects before conversion into the intermediate phase (**b**). **c**–**d** ADF-STEM images showing that line defects can be narrowing that is followed by forming the intermediate phase

Figure 3 shows a formation of an intermediate phase within a monolayer WS_2_ sheet, removing the line defect or narrowing its width. In Fig. 3a and b, a line defect disappears and leaves the intermediate phase through the migration of W and S atoms composed of the line defect. Although the line defect did not contact the edge of the extended hole, it was converted into the intermediate phase. In Fig. 3c and d, other line defects have been converted to the intermediate phases, which are evident by tracing white dashed arrows. This shows that even if line defects are formed inside the WS_2_ sheet, they can be converted to the intermediate phase.

**Fig. 4:**
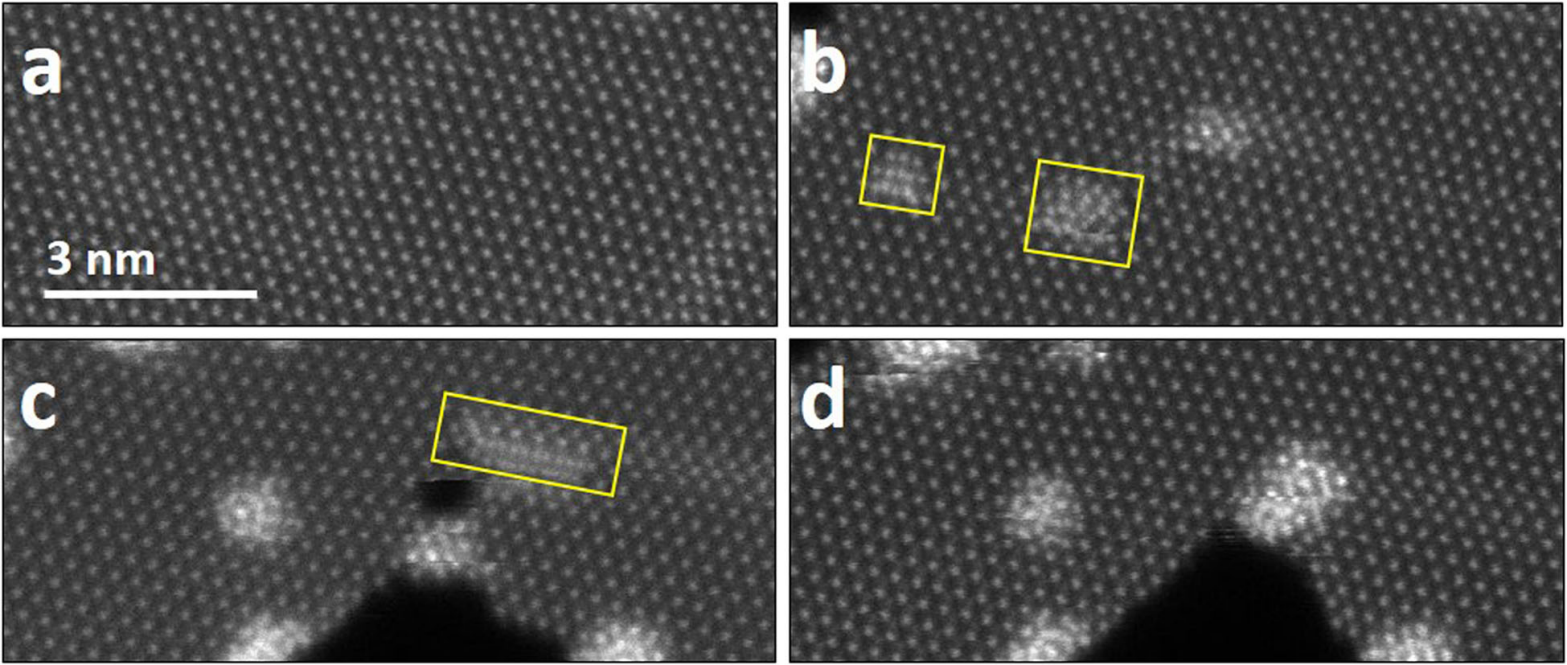
Transport of W atoms along line defects. **a** ADF-STEM image showing the WS_2_ sheet. **b**–**c** ADF-STEM images showing transport W atoms. Yellow boxes indicate transporting W atoms to form clusters. **d** Transition from the transporting atoms into the intermediate phase at the edge of the hole

We also observed the presence of some freely migrating W atoms in the WS_2_ sheet, due to the formation of line defects. For a hole to extend, both W and S atoms need to be ejected, but at low accelerating voltages, the knocking threshold of W is limited. This causes W atoms to accumulate at the edges of the hole and aggregate into clusters, which lowers the surface energy of this condition. Figure 4a shows the WS_2_ sheet before forming holes. Line defects linked to a hole allows W atoms to migrate along the line defect indicated by yellow boxes in Fig. 4b and c. The transport of W atoms occurs when linear defects interact with the hole. The line defect leads to the hole aggregating W atoms with the intermediate phase. Transporting W atoms along the line defects also is converted to intermediate phases at the edge of the hole (Fig. 4d).

**Fig. 5.**
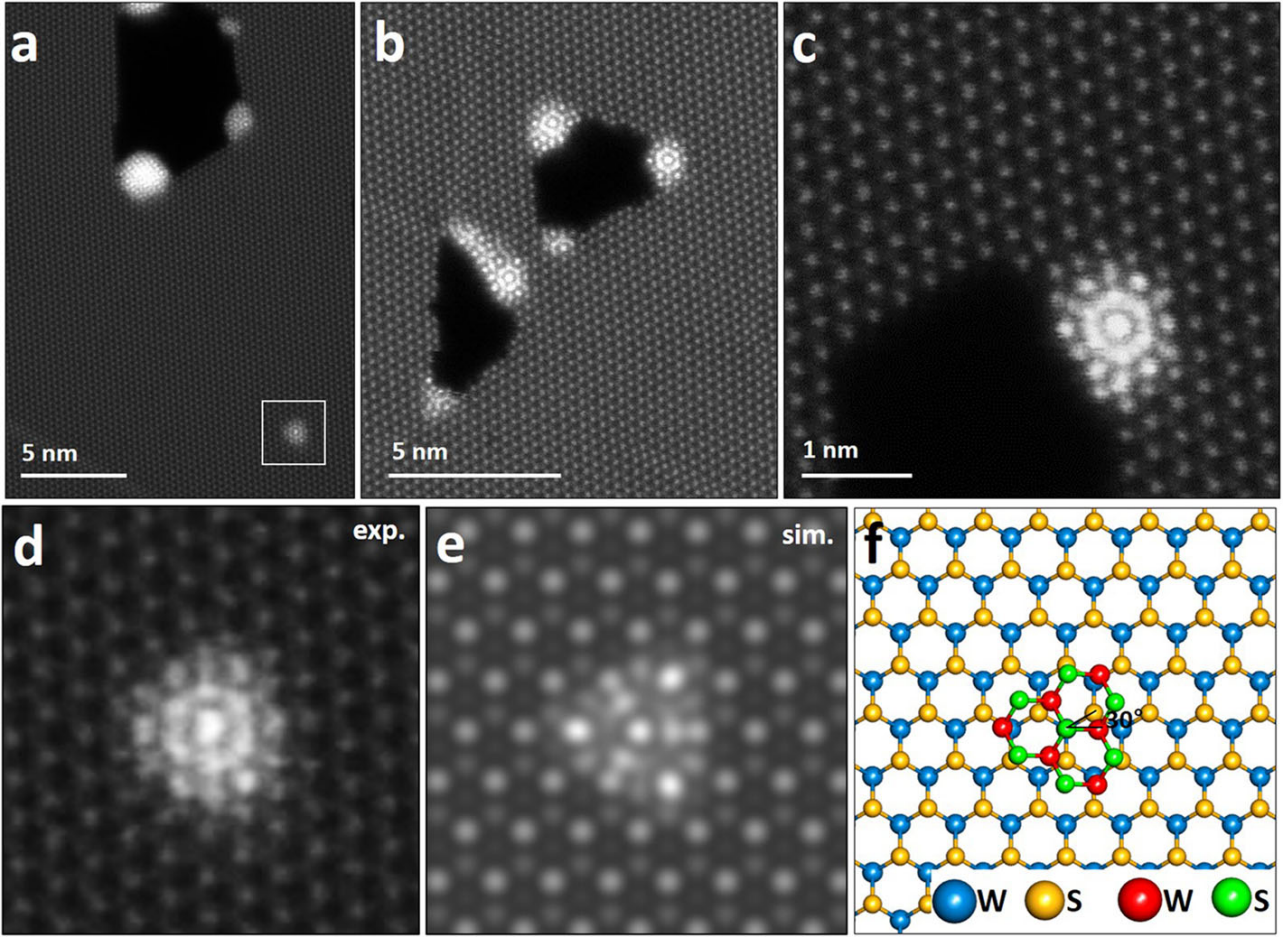
Atomic structures of the intermediate phase. **a** ADF-STEM image showing a hole with clusters and a intermedate phase positioned independently inside WS2 sheet. **b**–**c** ADF-STEM images showing intermediate phases at the edge of holes. **d** Magnified image of a white box in (**a**). **e** Image simulation of the intermedate phase according to an atomic model (**f**)

The entire processe explains the hole formation and its connection with line defects and the intermediate phase. When line defects are connected to a hole, S atoms are ejected from the edge region by migrating along the line defects. This creates local S exhaustion at the edge and the edge is reconstructed into WS nanowires or W clusters. Eventually, line defects disappear by migrating toward extended holes is directly followed by extension of the the holes. During this process, some W and S atoms composed of the line defects migrate and aggregate at the edge of the holes and inside the WS_2_ sheet, which is the end of the line defects or region where the line defects disappear. Here, the intermediate phase appears as shown in Fig. 5a-c. The intermediate phase has a uniform structure (Fig. 5d), which looks like a small sunflower-shaped. The W and S atoms composed of line defects migrate toward the hole and they reach the end of the line defects, where W clusters gradually grow on the WS_2_ surface. To analyze the atomic structure of the intermediate phase, image simulation was performed according to the atomic model (Fig. 5e and f). This is consistent with the hexagonal lattice of the rotated bilayer WS_2_, which is well matched with the experimental image of Fig. 5d, and the bright-contrast structures are due to a superposition of W and S atoms, with a rotation. As expected, the W cluster is mainly formed at the ends of the line defect contacting the holes, which leads to the phase on the WS_2_ sheet. The phase is fully converted into the W cluster by prolonged electron beam irradiation.

## Conclusions

We summarize that the line defects induced by electron beam irradiation migrate and diffuse into the holes, causing W atoms to aggregate at the hole edges of the monolayer WS_2_ sheet. When the S vacancies are formed individually, they are arranged linearly by forming long line defects. And then, holes are formed at the points where the line defects collide each other, and W atoms aggregate at the edges of the holes. The holes grow to extended holes that absorb line defects with further knocking out the W and S atoms, and this process occurs repeatedly. At this point, before the W atoms form a complete cluster, they go through the intermediate phase that has been analyzed as a uniform crystalline, and this phase interacts with the migration of the line defects. Complete W clusters usually form at the edge of the hole, but this intermediate phase can form not only at the edge of the hole, but also inside the WS_2_ sheet where line defects appeared. These results show the most detailed insights into the cluster formation mechanism and its association with topological defects activated in the TMD materials.

## Methods

### Synthesis and transfer

A double-walled quartz tube was inserted through two channels. S precursor powder (300 mg, 99.5%) was placed in an outer tube and aligned with the first furnace. The WO_3_ (200 mg, 99.9%) precursor was inserted into the second CVD tube inside the inner tube, the center of the hot zone of the heating furnace, and the substrate (Si/SiO_2_ chip) was placed on the outer tube. Pre-calibrated distance further downstream. The reaction vapor was brought to the substrate using an Ar carrier gas to allow WO_3_ sulfidation from the substrate. The first, S-containing furnace was kept at 180 °C, the second furnace was maintained at 1170 °C, and the reaction step took 3 min. The sample was rapidly cooled by removing from the furnace after the reaction step.

Transfer was performed by spin coating the sample with a supporting PMMA scaffold (8% wt, Mw 495 k). The PMMA/WS_2_ stack was separated from the SiO_2_/Si substrate by KOH etching (1 M) at 60 °C. The PMMA/WS_2_ film was transferred to clean glass slides through deionized water to rinse residue off the WS_2_ side and repeated several times. The film was then transferred to the sample chip, dried overnight, and then heated on a hot plate at 150 °C to evaporate remaining water and promote sample adhesion.

### Transmission electron microscopy

ADF-STEM was conducted using an aberration-corrected JEOL ARM200 STEM equipped with a JEOL corrector operated at an accelerating voltage of 80 kV located the David Cockayne Center. Dwell times of 5–20 μs and a pixel size of 0.006 nm px − 1 was used for imaging with a convergence semi-angle of 31.5 mrad, a beam current of 44 pA, and inner-outer acquisition angles of 49.5–198 mrad.

### Image processing and simulation

ImageJ was used to process the ADF images. Multislice image simulations for ADF images were performed using the multislice method implemented in the JEMS software.

## Data Availability

The datasets used and/or analyzed during the study are available from the corresponding author on reasonable request.

## Abbreviations

TMD: Transition metal dichalcogenides
ADF-STEM: Annular dark field scanning transition electron microscopy
0D: Zero dimensional
1D: One dimensional
2D: Two dimensional
